# Self-Related Processing and Deactivation of Cortical Midline Regions in Disorders of Consciousness

**DOI:** 10.3389/fnhum.2013.00504

**Published:** 2013-08-26

**Authors:** Julia Sophia Crone, Yvonne Höller, Jürgen Bergmann, Stefan Golaszewski, Eugen Trinka, Martin Kronbichler

**Affiliations:** ^1^Centre for Neurocognitive Research, Neuroscience Institute, Christian Doppler Klinik, Paracelsus Medical University, Salzburg, Austria; ^2^Department of Psychology and Centre for Neurocognitive Research, University of Salzburg, Salzburg, Austria; ^3^Department of Neurology, Christian Doppler Klinik, Paracelsus Medical University, Salzburg, Austria

**Keywords:** consciousness, vegetative state, self, anterior cingulate, default network

## Abstract

Self-related stimuli activate anterior parts of cortical midline regions, which normally show task-induced deactivation. Deactivation in medial posterior and frontal regions is associated with the ability to focus attention on the demands of the task, and therefore, with consciousness. Studies investigating patients with impaired consciousness, that is, patients in minimally conscious state and patients with unresponsive wakefulness syndrome (formerly vegetative state), demonstrate that these patients show responses to self-related content in the anterior cingulate cortex. However, it remains unclear if these responses are an indication for conscious processing of stimuli or are due to automatic processing. To shed further light on this issue, we investigated responses of cortical midline regions to the own and another name in 27 patients with a disorder of consciousness and compared them to task-induced deactivation. While almost all of the control subjects responding to the own name demonstrated higher activation due to the self-related content in anterior midline regions and additional deactivation, none of the responding patients did so. Differences between groups showed a similar pattern of findings. Despite the relation between behavioral responsiveness in patients and activation in response to the own name, the findings of this study do not provide evidence for a direct association of activation in anterior midline regions and conscious processing. The deficits in processing of self-referential content in anterior midline regions may rather be due to general impairments in cognitive processing and not particularly linked to impaired consciousness.

## Introduction

Self-related content is processed in several cortical midline regions (Kelley et al., 2002; Northoff and Bermpohl, 2004; Mitchell et al., 2005; Northoff et al., 2006; Uddin et al., 2007; Zhu et al., 2007; Platek et al., 2008; Yaoi et al., 2009; Herwig et al., 2012; Salomon et al., 2013). Most of these studies involve an evaluation of self-related in comparison to other content which is interpreted as a differentiation between both, and therefore, as conscious awareness of self. However, self-related stimuli, in contrast to only familiar and other stimuli, activate anterior parts of the default mode network (DMN) such as the anterior cingulate cortex (ACC) (Qin and Northoff, 2011). These regions normally show deactivation during tasks involving higher cognitive and attention-demanding processing of external stimuli (Shulman et al., 1997b; Greicius and Menon, 2004). It is postulated that deactivation corresponds to an interruption of internal ongoing processes to make resources available that are necessary to focus attention on the demands of the task (Gusnard et al., 2001; Raichle and Snyder, 2007; Anticevic et al., 2012). Focusing attention to solve cognitive tasks is a process that goes along with conscious awareness of the environment (Fransson, 2005). While other attention-demanding stimuli interrupt the activity in the ACC (Shulman et al., 1997a), self-related stimuli do not (Qin and Northoff, 2011). Qin and Northoff (2011) speculate that self-related processing may be present not only during conscious awareness of external stimuli but during resting state itself.

Investigations of preserved brain responses to self-related stimuli such as the own name have been performed in subjects with reduced or impaired conscious awareness. Patients with unresponsive wakefulness syndrome (formerly vegetative state; VS/UWS) and in minimally conscious state (MCS), i.e., patients with a disorder of consciousness (DOC) after severe brain injury, are, by definition, not or only minimal consciously aware. Diagnosis in these patients is still very challenging (Schnakers et al., 2009), and thus, several attempts have been made to find additional diagnostic criteria linking brain responses to conscious behavior (e.g., Laureys et al., 2002; Boly et al., 2004; Owen et al., 2006; Monti et al., 2010; Schnakers et al., 2010; Fernandez-Espejo et al., 2011; Goldfine et al., 2011; Gosseries et al., 2011; Naci et al., 2012; Estraneo et al., 2013). Self-relatedness has been of particular interest because studies could demonstrate corresponding brain responses in these patients (Mazzini et al., 2001; Kotchoubey et al., 2004; Laureys et al., 2004; Perrin et al., 2006; Di et al., 2007; Schnakers et al., 2008). A single-subject study, for example, investigating responsiveness to the own name in a patient diagnosed as VS/UWS detected preserved activation in cortical midline structures (Staffen et al., 2006). Another study in seven VS/UWS and four MCS patients demonstrates that the ACC in particular is responsive to self-related stimuli in patients diagnosed as unconscious or minimally conscious (Qin et al., 2010). Moreover, this responsiveness correlates with the level of behavioral responses the patient is able to perform. The authors propose that neural activity in the ACC during self-relatedness may be a diagnostic marker for the degree of consciousness in patients. Yet, the authors themselves emphasize that activation of the ACC may only reflect automatic processing of self-related stimuli rather than conscious processes. Since activity of the ACC is present during resting state and this region is normally suppressed in response to conscious processing of external stimuli, and since it is not known to which extent conscious awareness of external stimuli is reflected in the resting brain, activity of the ACC in response to self-related content does not necessarily reflect conscious processing. Moreover, self-related speech was not directly compared to non-self-related speech in the previous study. But especially for diagnostic criteria, it is essential to find evidence for conscious processing and to exclude the possibility that the association with the degree of behavioral responsiveness is rather due to a more general deficit in cognitive processing of association areas.

So far, previous studies whether performed in healthy subjects or in impaired consciousness could not sufficiently clarify the relationship between activation in anterior cortical midline regions in response to self-related stimuli and conscious processing. The aim of this study is twofold: first, we want to extend previous findings in patients with DOC by including a control condition for self-referential processing and second, we want to take into account additional brain responses which may provide further indication for conscious processing of stimuli. A recent study in patients with DOC was able to show that listening to sentences induces deactivation in all healthy and thus conscious subjects but only in 9 out of 25 patients in regions of the DMN (Crone et al., 2011). Eight of these patients also showed activity in response to language in frontal regions associated with conscious processing. The conclusion is that deactivation of the DMN seems to reflect conscious and attention-involved processing of external stimuli.

Based on the study by Qin et al. (2010), we investigated activation in response to the own name in impaired consciousness with two important improvements: we included a control condition for self-relatedness to be able to associate findings specifically to self-related processing and we looked for deactivation in regions of the DMN during stimuli processing to identify possible indicator for conscious processing. First, we want to see if processing of the own name in the ACC in patients is related to the self-referential aspect of the own name tested with a control condition. Second, we want to search for other indications of conscious processing in patients showing responses to the self-related content. If responding to the own name compared to another goes along with deactivation and non-responding with no deactivation, this may support the assumption that activity in the ACC during self-relatedness can be associated with consciousness. If self-related content, though, is processed in DOC patients without a disruption of internal processes within the DMN while healthy controls show both, it remains questionable if responses of the ACC to self-related content are a valid marker for the degree of consciousness.

## Materials and Methods

The study was approved by the Ethical Committee of Salzburg (Ethics Commission Salzburg/Ethikkommission Land Salzburg; number 415-E/952).

### Subjects

In this study, 17 healthy subjects, 21 patients with VS/UWS, and 9 patients in MCS were investigated. Three patients had to be excluded from further analysis due to severe head motion (translation ≥2.5 mm; rotation ≥2.5°). The remaining 18 patients with VS/UWS (mean age = 50; 6 female) and 9 patients in MCS (mean age = 47; 5 female) were compared to 17 healthy subjects (mean age = 44; 10 female). Patients were clinically assessed once a week during in-patient stay using standardized scales, i.e., the Coma Recovery Scale-Revised (CRS-R) (Giacino et al., 2004). All patients participating in this study showed preserved auditory functioning, largely preserved brainstem reflexes, and a fairly preserved sleep-wake-cycle based on neurological examination. None of the patients were artificially ventilated or sedated at time of scanning. Additional information of the patients is listed in Table 1. Control subjects were recruited at the Paris Lodron University of Salzburg. Written informed consent was obtained from all healthy subjects and from the guardianship of all patients according to the Declaration of Helsinki.

**Table 1:** **Patients’ information; MCS, minimally conscious state; VS/UWS, unresponsive wakefulness syndrome**.

Subjects	Sex	Age	Etiology	Time since onset (in days)	Auditory function	Visual function	Motor function	Verbal/oromotor function	Communication	Arousal
**MCS**										
MCS01	F	45	Traumatic brain injury	2960	Auditory startle	Fixation	Flexion withdrawal	Oral reflexive movement	None	With stimulation
MCS02	F	54	Subarachnoidal hemorrhage	640	Auditory startle	Fixation	Flexion withdrawal	Oral reflexive movement	None	With stimulation
MCS03	M	51	Intracerebral Hemorrhage	102	Localization to sound	Visual pursuit	Abnormal posturing	Oral reflexive movement	None	Without stimulation
MCS04	M	37	Respiratory failure	67	Auditory startle	Fixation	Flexion withdrawal	Oral reflexive movement	None	Without stimulation
MCS05	M	47	PICA infarct	49	Auditory startle	Fixation	Abnormal posturing	Oral reflexive movement	Intentional	Without stimulation
MCS06	M	47	Traumatic brain injury	52	Localization to sound	Fixation	Abnormal posturing	Oral reflexive movement	None	Without stimulation
MCS07	F	55	Subarachnoidal hemorrhage	277	Auditory startle	Visual pursuit	Flexion withdrawal	Oral reflexive movement	None	Without stimulation
MCS08	F	28	Limbic encephalopathy	154	None	Visual pursuit	Flexion withdrawal	Oral reflexive movement	None	Without stimulation
MCS09	F	63	Subarachnoidal hemorrhage	62	Auditory startle	Object localization: reaching	Flexion withdrawal	None	None	Without stimulation
**UWS**										
VS/UWS01	M	45	Cardiopulmonary resuscitation	635	Auditory startle	None	None	Oral reflexive movement	None	With stimulation
VS/UWS02	M	50	Cardiopulmonary resuscitation	204	Auditory startle	None	Flexion withdrawal	Oral reflexive movement	None	Without stimulation
VS/UWS03	M	69	Cardiopulmonary resuscitation	58	Auditory startle	None	Flexion withdrawal	Oral reflexive movement	None	Without stimulation
VS/UWS04	F	39	Respiratory failure	73	Auditory startle	Visual startle	Abnormal posturing	None	None	With stimulation
VS/UWS05	M	47	Cardiopulmonary resuscitation	65	Localization to sound	Visual startle	Flexion withdrawal	Oral reflexive movement	None	Without stimulation
VS/UWS06	M	45	Traumatic brain injury, mediainfarct	182	Auditory startle	None	Flexion withdrawal	None	None	With stimulation
VS/UWS07	F	29	Basilaris thrombosis	104	Auditory startle	None	Flexion withdrawal	Oral reflexive movement	None	With stimulation
VS/UWS08	M	78	Cardiopulmonary resuscitation	39	Auditory startle	None	None	Oral reflexive movement	None	With stimulation
VS/UWS09	F	47	Multiple ischemic infarct	51	Auditory startle	None	Flexion withdrawal	Oral reflexive movement	None	With stimulation
VS/UWS10	M	63	Cerebral hypoxia	16	Auditory startle	None	Abnormal posturing	Oral reflexive movement	None	With stimulation
VS/UWS11	M	51	Cardiopulmonary resuscitation	30	None	None	None	Oral reflexive movement	None	With stimulation
VS/UWS12	M	50	Ischemic brainstem infarct	165	Auditory startle	None	Flexion withdrawal	Oral reflexive movement	None	With stimulation
VS/UWS13	F	51	Cardiopulmonary resuscitation	1471	Auditory startle	None	Flexion withdrawal	Oral reflexive movement	None	Without stimulation
VS/UWS14	F	49	Cardiopulmonary resuscitation	40	Auditory startle	None	Flexion withdrawal	Oral reflexive movement	None	Without stimulation
VS/UWS15	F	38	Subarachnoidal hemorrhage	327	Auditory startle	None	Flexion withdrawal	Oral reflexive movement	None	Without stimulation
VS/UWS16	M	61	Traumatic brain injury	116	None	None	None	Oral reflexive movement	None	Without stimulation
VS/UWS17	M	35	Cardiopulmonary resuscitation	38	None	None	Flexion withdrawal	Oral reflexive movement	None	With stimulation
VS/UWS18	M	47	Cardiopulmonary resuscitation	3629	Auditory startle	None	Abnormal posturing	Vocalization/oral movement	None	Without stimulation

### Data acquisition

Control subjects and patients were scanned while listening to their own name or another name (e.g., Martin, hello Martin). Stimuli were recorded in German language with Cool Edit Pro 2.00 (1992–2000 Syntrillium Software Corporation) by two men and two women, none of which were familiar to the patient or knew his real first name. Two fMRI sessions were performed, each containing 30 stimuli of the own name and 30 stimuli of the other name, as well as 30 silent null events (duration = 2200 ms; ISI = 1800 ms). Stimuli were presented in an event-related design in pseudo-randomized order. During each run, 180 functional images were acquired using a 3T Philips scanner (Philips Achieva; 21 slices with a thickness of 6 mm; matrix size = 64 × 64; FoV = 210 mm^2^; TR = 2200 ms; TE = 45 ms; flip angle = 90°) and a 3T Siemens scanner (Siemens TIM TRIO; 21 slices with a thickness of 6 mm; matrix size = 80 × 80; FoV = 210 mm^2^; TR = 2200 ms; TE = 30 ms; flip angle = 70°). Eight control subjects, five patients in MCS, and 11 patients with VS/UWS were investigated with the Philips Achieva and seven control subjects, four patients in MCS, and seven patients with VS/UWS were investigated with the Siemens TIM TRIO. In addition, high-resolution, T1-weighted MPRAGE sequences for anatomic information were acquired for each participant.

### Data analyses

Functional data were preprocessed and analyzed using Statistical Parametric Mapping (version SPM8; Wellcome Department of Cognitive Neurology, London, UK)^1^. The first six functional scans were considered as dummy scans and were discarded. For this investigation, we performed a group analysis and a single-subject analysis. Both analyses are important because results at the group level do not always reflect findings in the single subject (Kotchoubey et al., 2004; Holler et al., 2011) which are crucial for diagnosis in patients with DOC. Thus, we implemented two different preprocessing approaches: for group analysis, preprocessing steps included the following procedures: segmentation of the T1 image to compute the gray matter images; realignment to compensate for motion; unwarping (adjustment for movement-related artifacts); pre-coregistration of the functional images of session 2 to session 1; coregistration of the mean EPI to the participant’s gray matter image; normalization of an average image of the functional images with the segmentation parameters; data were spatially smoothed using a Gaussian Kernel of 8 mm full width at half maximum (FWHM); For single-subject statistical analysis, we did not perform normalization of the functional data to avoid artifacts induced by severe lesions. Voxel-wise statistical parametric maps were generated for each subject. Both conditions (own name and another name) and six realignment parameters were included in the model. The data were high-pass filtered with a cutoff at 128 s and corrected for serial correlations.

To extend findings of the previous study in patients with DOC by Qin et al. (2010), we performed a ROI analysis at the single-subject level with the contrast own name vs. rest. A second contrast, own name vs. another, was applied to relate findings specifically to self-relatedness. Additionally, a third contrast, another name vs. rest, was selected to investigate deactivation in cortical midline regions.

To show differences between the three levels of consciousness (healthy controls, MCS, and VS/UWS), we performed a group analysis. Subject-specific contrast images were entered into a voxel-based second level analysis. Differences between groups were computed with an ANCOVA with group as a factor. For *post hoc* testing *t*-tests were applied. To address the problem of possible confounds of the two types of scanners and the differences in mean age between groups, we included scanner type and age each as a covariate.

The ROI analysis was performed for each cortical midline region using a small volume with a sphere of 10 mm radius. ROIs for responses to self-related content were chosen according to the study by Qin et al. (2010). In this study, three main ROIs in anterior medial cortical areas were identified for self-related processing validated in two experiments with healthy subjects: the caudal part of the ACC (cACC; 10, 18, 36); the supplementary motor area (SMA; 0, 13, 59); the anterior part of the ACC (aACC; 1, 26, 19). ROIs within the cortical midline structures for deactivation in response to another name were chosen from a large meta-analysis of DMN functional heterogeneity by Laird et al. (2009): precuneus (−2, −56, 50); posterior cingulate cortex (PPC; −5, −52, 25), medial prefrontal cortex (MPFC; −1, 55, 8). Coordinates were selected as specified in both publications and transformed into Montreal Neurological Institute (MNI) space for the group analysis. For single-subject analysis, the inverse of the normalization parameters were used to warp the ROI images to the particular image of each subject. Additionally, a Pearson correlation analysis (two-tailed) was performed between the mean contrast estimates from each ROI in all patients and the scores of the CRS-R to relate brain responses to behavioral responses and cognitive functioning. Correlation analyses were computed with SPSS (version 14; SPSS inc.)^2^. ROI analyses were corrected for FWE at the voxel level with a threshold of *p* < 0.05.

To address the ongoing debate of possible effects of small head motion on group comparisons, we excluded all sessions of subjects with head motion above a defined criterion and ensured that there were no differences between the three groups in any of the motion parameters by calculating a One-way ANOVA with group as a factor (*F* ≤ 1.77, *p* ≥ 0.185).

## Results

### ROI analyses at group level

#### Group results

In response to the own name vs. rest, significant activation in the control group at a corrected level was found in two ROIs: in the SMA (*t* = 4.92, *p* = 0.007) and in the cACC (*t* = 3.87, *p* = 0.049). The MCS group showed significant activation in the aACC (*t* = 6.36, *p* = 0.033). The VS/UWS group had no significant activation. Uncorrected, significant activation was found in the SMA for the MCS group (*t* = 2.22, *p*_uncorr_ = 0.013) and for the VS/UWS group (*t* = 2.49, *p*_uncorr_ = 0.006).

In response to the own vs. another name, significant activation was found only at the uncorrected threshold level: the control group showed significant activation in the SMA (*t* = 2.58, *p*_uncorr_ = 0.005) and in the cACC (*t* = 2.69, *p*_uncorr_ = 0.008). The MCS group exhibited significant activation in the cACC (*t* = 1.75, *p*_uncorr_ = 0.040).

Significant deactivation was found in the precuneus (*t* = 7.93, *p* < 0.001), in the PCC (*t* = 5.09, *p* = 0.011), and in the MPFC (*t* = 4.29, *p* = 0.024) for the controls at a corrected level. Both patient groups did not show any significant deactivation, neither corrected nor uncorrected.

#### Differences between groups

**Figure 1:**
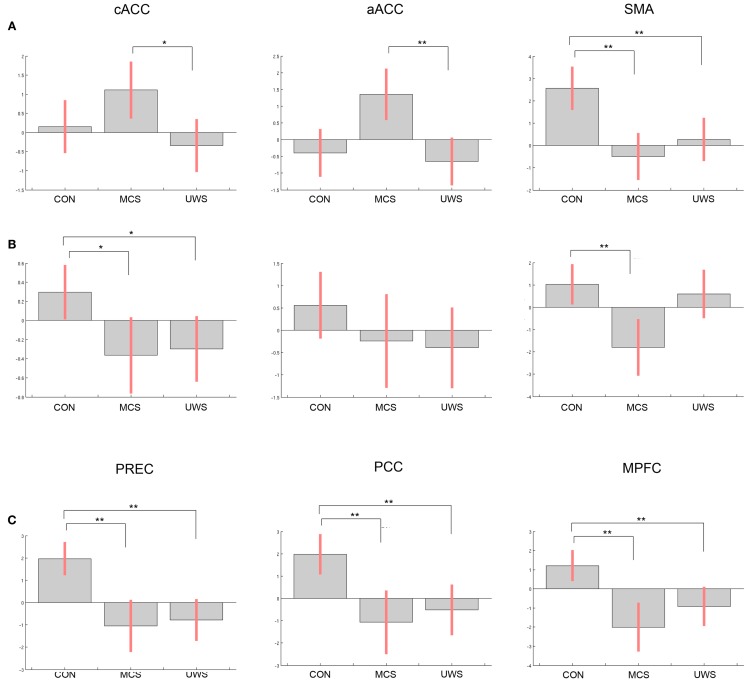
Differences of contrast estimates between healthy controls, patients in minimally conscious state, and patients with unresponsive wakefulness syndrome in the regions of interest (ROI) for (A) own name vs. rest, (B) own name vs. another name, (C) deactivation in response to another name vs. rest (note that deactivation is shown with positive values); bars display contrast estimates at the center of the ROI and 90% confidence interval; CON, controls; MCS, minimally conscious state; VS/UWS, unresponsive wakefulness syndrome; cACC, caudal part of the anterior cingulate cortex; aACC, anterior part of the anterior cingulate cortex; SMA, supplementary motor area; PREC, precuneus; PCC, posterior cingulate cortex; MPFC, medial prefrontal cortex; significant differences between groups are indicated with **p < 0.05, corrected for family-wise error, and *p < 0.05, uncorrected.

Significant differences between control subjects and patients were evident in processing of the own name and another name (see Figure 1).

Differences in response to own name vs. rest were significant in the SMA between healthy controls and patients in MCS (*t* = 3.53, *p* = 0.006) and between controls and patients in VS/UWS (*t* = 2.94, *p* = 0.030). Comparing MCS with VS/UWS, differences were significant in the aACC (*t* = 3.19, *p* = 0.050), and at an uncorrected threshold level in the cACC (*t* = 2.95, *p*_uncorr_ = 0.003).

In response to the own name vs. another, differences were significant in the SMA between control subjects and MCS (*t* = 3.04, *p* = 0.023). Uncorrected, additional significant differences were found in the cACC for controls vs. MCS (*t* = 1.77, *p*_uncorr_ = 0.038) and for controls vs. UWS (*t* = 2.53, *p*_uncorr_ = 0.008).

Differences in deactivation were significant between controls and MCS in the precuneus (*t* = 5.57, *p* < 0.001), in the PCC (*t* = 4.46, *p* = 0.002), and in the MPFC (*t* = 4.57, *p* = 0.002). Between controls and VS/UWS differences were significant in the precuneus (*t* = 6.75, *p* < 0.001), in the PCC (*t* = 4.16, *p* = 0.005), and in the MPFC (*t* = 3.89, *p* = 0.009). There were no significant differences between the patient groups neither at an uncorrected threshold level nor corrected for multiple comparisons.

### ROI analyses at single-subject level

**Figure 2:**
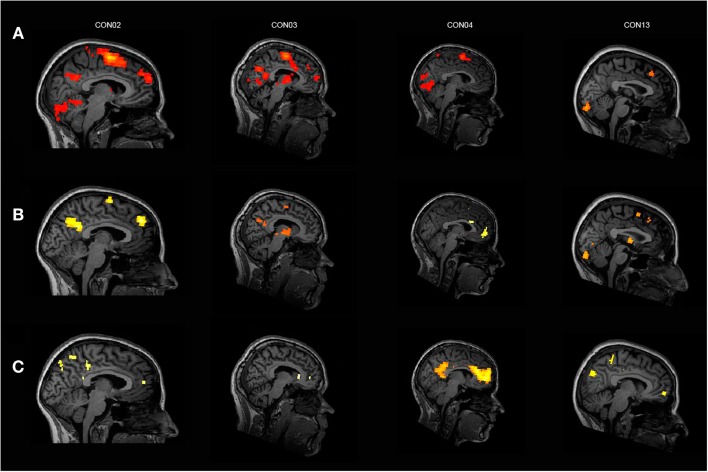
**Four healthy control subjects showing (A) significant activation in response to the own name vs. rest, (B) significant activation in response to the own name vs. another name, and (C) significant deactivation in response to another name vs. rest; for display purposes, results are thresholded at *p* < 0.001 at the whole brain level, uncorrected for multiple comparisons**.

Almost all of the control subjects, showing activation in response to the own name in one or more ROIs, deactivated in response to another name in at least one of the three ROIs except for two subjects who only showed significant activation in the SMA. Figure 2 displays four healthy control subjects showing responses in the selected ROIs. Four controls deactivated in one or more of the corresponding ROIs without responding to the own name. One patient in MCS showed activation in the aACC but no deactivation. Additionally, three patients with VS/UWS showed activation in one ROI (two in the SMA; one in the cACC and aACC) but also no deactivation. Most of the control subjects exhibiting activation in response to the own name showed additional significant higher activation in response to the own name when directly compared to another name. Only one patient showed higher activation in response to the own name vs. another but without responding to the own name vs. rest, however. See Table 2 for detailed information.

**Table 2:** **Significant activation in response to the own name and to the own name vs. another in regions of interest at the single-subject level; *t*-values are shown corrected for family-wise error at voxel level with *p* < 0.05; CON, controls; MCS, minimally conscious state; VS/UWS, unresponsive wakefulness syndrome; cACC, caudal part of the anterior cingulate cortex; aACC, anterior part of the anterior cingulate cortex; SMA, supplementary motor area; PREC, precuneus; PCC, posterior cingulate cortex; MPFC, medial prefrontal cortex**.

Subject	cACC		aACC		SMA		PREC	PCC	MPFC
				
	Own > rest	Own > other	Own > rest	Own > other	Own > rest	Own > other	Other < rest		
CON01	–	–	–	–	–	–	–	–	–
CON02	3.45	–	–	–	9.19	–	3.45	3.52	2.71
CON03	4.43	2.95	3.55	–	6.84	4.24	–	–	2.86
CON04	–	3.10	–	3.74	5.79	3.22	–	5.74	6.32
CON05	–	–	–	–	5.04	–	–	–	2.97
CON06	–	–	–	–	6.48	4.24	–	–	–
CON07	–	–	–	–	–	–	–	–	–
CON08	–	–	–	–	–	–	–	–	–
CON09	–	–	–	–	–	–	4.38	3.42	–
CON10	–	–	–	–	6.43	4.28	–	–	–
CON11	–	–	–	–	–	–	–	–	–
CON12	–	–	–	–	–	–	4.37	5.33	5.74
CON13	–	3.08	–	–	3.64	4.08	3.04	–	–
CON14	–	–	–	–	–	–	3.94	–	–
CON15	–	–	–	–	–	–	–	–	–
CON16	–	–	–	–	–	–	–	–	–
CON17	–	–	–	–	–	–	4.35	–	–
MCS01	–	–	–	–	–	–	–	–	–
MCS02	–	–	3.03	–	–	–	–	–	–
MCS03	–	–	–	–	–	–	–	–	–
MCS04	–	–	–	–	–	–	–	–	–
MCS05	–	–	–	–	–	–	–	–	–
MCS06	–	–	–	–	–	–	–	–	–
MCS07	–	–	–	–	–	–	–	–	–
MCS08	–	–	–	–	–	–	–	–	–
MCS09	–	–	–	–	–	–	–	–	–
VS/UWS01	–	–	–	–	–	–	–	–	–
VS/UWS02	–	–	–	–	–	–	–	–	–
VS/UWS03	–	–	–	–	–	–	–	–	–
VS/UWS04	–	–	–	–	–	–	–	–	–
VS/UWS05	3.33	–	3.31	–	–	–	–	–	–
VS/UWS06	–	–	–	–	3.25	–	–	–	–
VS/UWS07	–	–	–	–	–	–	–	–	–
VS/UWS08	–	–	–	–	–	–	–	–	–
VS/UWS09	–	–	–	–	–	2.94	–	–	–
VS/UWS10	–	–	–	–	–	–	–	–	–
VS/UWS11	–	–	–	–	–	–	–	–	–
VS/UWS12	–	–	–	–	–	–	–	–	–
VS/UWS13	–	–	–	–	–	–	–	–	–
VS/UWS14	–	–	–	–	–	–	–	–	–
VS/UWS15	–	–	–	–	–	–	–	–	–
VS/UWS16	–	–	–	–	–	–	–	3.09	–
VS/UWS17	–	–	–	–	3.34	–	–	–	–
VS/UWS18	–	–	–	–	–	–	–	–	–

### Additional analyses

**Figure 3:**
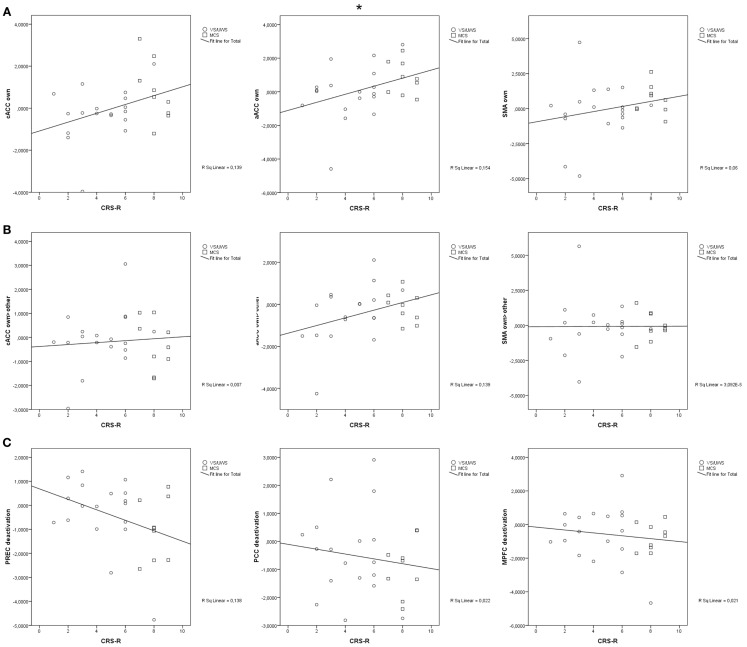
**(A)** significant correlation of activation in response to the own name in anterior midline regions; **(B)** significant correlation of activation in response to the own name vs. another name in anterior midline regions; **(C)** significant correlation of deactivation in response to another name in posterior and frontal midline regions; cACC, caudal part of the anterior cingulate cortex; aACC, anterior part of the anterior cingulate cortex; SMA, supplementary motor area; PREC, precuneus; PCC, posterior cingulate cortex; MPFC, medial prefrontal cortex; CRS-R, Coma Recovery Scale-Revised; *p < 0.05.

Correlations between the behavioral scores of the CRS-R and responses to the own name were significant in the aACC (*r* = 0.39, *p* = 0.043). There were no significant correlations between the CRS-R scores and responses to own vs. another name in any anterior region (cACC: *r* = 0.09, *p* = 0.670; aACC: *r* = 0.37, *p* = 0.055; SMA: *r* = 0.01, *p* = 0.978). The correlation between the scores and deactivation in response to another name were not significant (precuneus: *r* = −0.37, *p* = 0.056; PCC: *r* = −0.15, *p* = 0.46; MPFC: *r* = −0.15, *p* = 0.467). Figure 3 displays all correlations.

To assess the relation between activation in response to the own name in general and response to self-related content in particular, Yates’ chi-square goodness of fit test was calculated assessing the number of subjects showing activation in response to the own name and to the own name vs. another in at least one ROI compared to those responding to only one of the contrasts or none [for the controls, χ^2^(1) = 6.97, *p* = 0.008, and for the patients, χ^2^(1) = 1.02, *p* = 0.313].

To exclude the possibility that a lack of activation in anterior midline regions is only due to a general absence of auditory processing in lateral temporal regions, we performed another Yates’ chi-square goodness of fit test for the patients, χ^2^(1) = 0.05, *p* = 0.818.

## Discussion

In this study, the brain response of 27 patients with DOC during self-referential processing was investigated and compared to deactivation in regions of the DMN.

At group level, we found significant activation of anterior midline regions in response to the own name in healthy controls and in one of the ROIs selected (aACC) in the group of subjects showing minimal signs of consciousness. In this region, we also found significant differences between both patient groups. Correspondingly, the degree of behavioral responsiveness in patients was related to the activation level in the aACC. These findings are in line with the study by Qin et al. (2010) which demonstrated that the ACC is involved in linking the self and consciousness.

In contrast to the study by Qin et al. (2010) though, a minority of patients demonstrated activation in response to the own name. Only 1 of the 9 MCS patients and 3 of the 18 VS/UWS patients showed a response in the selected ROIs. The possibility that lack of activation of the ACC may be due to a general lack of auditory processing can be excluded since there was no association between lack of activation in anterior midline regions and lack of activation in lateral temporal areas in patients.

Furthermore, the correlation between the scores of the CRS-R and the contrast estimates extracted from each ROI was only very weak in the ACC while in the population investigated by Qin et al. the correlation was very strong. These differences are an important finding because they demonstrate the high variability in patients with severe head injury perhaps due to the differences in cause, location, and dimension of the injuries. This corresponds with a previous study demonstrating that the etiology may influence brain responses stronger than the degree of consciousness (Fischer et al., 2010). Especially when investigating such a small number of patients as in the study by Qin et al. (four MCS and seven VS/UWS), this may be of particular relevance. Apart from that, it is important to note that we did not use a block-design to present the stimuli which may contribute to the reduced responsiveness. We also used a sphere of 10 mm for all ROIs. The size and form of the ROIs may influence the results as well.

A limitation of the study by Qin et al. (2010) is that they did not implement a control condition for the patients. To relate the activation observed in patients to self-referential processing directly, we included the contrast own name vs. another name in our analysis. Comparing the groups revealed significant lower responsiveness for patients to self-related stimuli in the cACC and SMA which is in line with the findings by Qin et al. But when examining the results at a single-subject level, it becomes evident that responses to the own name go along with responsiveness to self-relatedness only in healthy subjects and not in patients. Moreover, there are no differences between the patient groups when comparing self-related to non-self-related content. Consistently, the activation of the aACC in the MCS group did not exceed the threshold for correction of multiple comparisons when directly associated with self-referential processing (own name vs. other). Thus, the observed responses to the own name in the selected ROIs may not necessarily be due to processing of self-related content in patients with severe head injury.

Another aim of this study was to find further indication for conscious processing of self-relatedness in anterior midline regions. While our findings endorse the conclusion of Qin et al. (2010) that sub-regions of the ACC are linking self and behavioral responsiveness to some extent, this link does not necessarily rely on conscious processing. Although the activation in response to the own name was related to the behavioral responsiveness of the patients in sub-parts of the ACC, the findings overall do not provide evidence for a direct association with consciousness. There were significant differences in deactivation between the control group and the patient groups in all three selected ROIs. Deactivation in regions of the DMN is present in tasks requiring higher cognition and attention-focusing (Shulman et al., 1997b; Greicius and Menon, 2004). None of the responding patients were able to interrupt ongoing internal processes to focus attention which would have been a further indication for conscious processing. None of the responding patients differentiated between the own name and another name. Consistently, the patient groups did not differ in their response to the own name compared to another. Moreover, the correlation between behavioral responsiveness and activation in response to the own name, as becomes evident from Figure 3, was very weak. The differences between the patient groups probably rather reflect the impairment of cognitive processing in general than the degree of conscious processing of the stimuli. The impact of brain injury seems to interfere with self-related processing in anterior midline regions to a similar extent as it does with processes of other higher association areas going along with deficits in consciousness (e.g., Di et al., 2007). To further prove this suggestion, it might be useful to compare stimuli processing in the ACC to processing in other brain areas, such as the auditory cortex.

Interestingly, the SMA demonstrated the strongest responses in healthy controls and the strongest deficits in processing of self-referential stimuli in patients. This area within the SMA (or the posterior part of the medial frontal cortex) is also known as a region involved in task control and attention monitoring (Amodio and Frith, 2006). An explanation might be that the own name with its self-related content in comparison to another name is much more involved in processes of attention which are highly affected in patients.

It is important to note that not all controls responding to the own name in the ROI analysis showed deactivation. Two of the control subjects activated in the SMA but did not deactivate in any of the frontal or posterior regions within the DMN.

Furthermore, not all healthy controls responded to the own name in anterior midline regions. However, this is not a very exceptional finding since previous EEG studies also found a high variability in responses to the own name at single-subject level (Kotchoubey et al., 2004; Holler et al., 2011).

A limitation of this study is that consciousness is sufficient but not necessary for deactivation of the DMN and that the absence of deactivation does not necessarily imply an absence of consciousness. Moreover, the participants listened to names instead of sentences as used in the previous study (Crone et al., 2011), which does not include processing of semantic knowledge. Therefore, these conclusions are limited to interpretation and further studies are required to confirm these findings.

In summary, this investigation demonstrates the high variability of responsiveness in severe brain injury and the need for replications in large patient populations. Additionally, it provides further indications that processing of self-related stimuli such as the own name in anterior midline regions does not necessarily reflect a conscious response to external stimuli in the sense of understanding and differentiating. While almost all of the conscious subjects responding to the own name showed higher activation to self-referential stimuli and demonstrated additional deactivation in medial posterior and frontal regions, none of the responding subjects with impaired consciousness did so. Although processing of self-related content in the ACC seems to require a certain level of cognitive functioning, it is questionable whether activation in response to self-related content in cortical midline regions directly reflects conscious processing. Instead, the observed deficits in patients may rather be associated with alterations of network structures which interfere with higher cognitive processing in general (see Corbetta, 2012 for review) and are additionally accompanied by a breakdown of consciousness.

## Conflict of Interest Statement

The authors declare that the research was conducted in the absence of any commercial or financial relationships that could be construed as a potential conflict of interest.

## Footnotes

^1^http://www.fil.ion.ucl.ac.uk/spm/

^2^www.spss.com
